# Cross-codal integration of bridging-event information in narrative understanding

**DOI:** 10.3758/s13421-020-01039-z

**Published:** 2020-04-27

**Authors:** Markus Huff, Dina Rosenfelder, Maria Oberbeck, Martin Merkt, Frank Papenmeier, Tino G. K. Meitz

**Affiliations:** 1 Knowledge Exchange Lab, Leibniz-Institut für Wissensmedien, Schleichstr. 6, D-72076 Tübingen, Germany; 2 Department of Psychology, Eberhard Karls Universität Tübingen, Tübingen, Germany; 3 Department Teaching, Learning, Counselling, German Institute for Adult Education, Bonn, Germany; 4 Department of Communication, University of Münster, Münster, Germany

**Keywords:** Bridging inferences, Narrative understanding, Cross-codal integration

## Abstract

Visual narratives communicate event sequences by using different code systems such as pictures and texts. Thus, comprehenders must integrate information from different codalities. This study addressed such cross-codal integration processes by investigating how the codality of bridging-event information (i.e., pictures, text) affects the understanding of visual narrative events. In Experiment 1, bridging-event information was either present (as picture or text) or absent (i.e., not shown). The viewing times for the subsequent picture depicting the end state of the action were comparable within the absent and the text conditions. Further, the viewing times for the end-state picture were significantly longer in the text condition as compared to the pictorial condition. In Experiment 2, we tested whether replacing bridging-event information with a blank panel increases viewing times in a way similar to the text condition. Bridging event information was either present (as picture) or absent (not shown vs. blank panel). The results replicated Experiment 1. Additionally, the viewing times for the end-state pictures were longest in the blank condition. In Experiment 3, we investigated the costs related to integrating information from different codalities by directly comparing the text and picture conditions with the blank condition. The results showed that the distortion caused by the blank panel is larger than the distortion caused by cross-codal integration processes. Summarizing, we conclude that cross-codal information processing during narrative comprehension is possible but associated with additional mental effort. We discuss the results with regard to theories of narrative understanding.

## Introduction

**Fig. 1:**
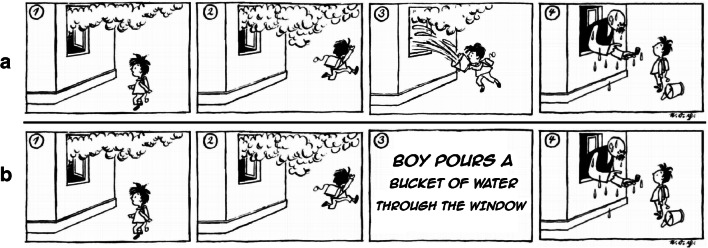
(**A**) Sequential, four-panel comic strip “Vater und Sohn [Father and Son]” (Ohser, 1962). (**B**) Modified version by the authors. While comics typically don’t replace pictures with words in such a dramatic way, (B) illustrates that narrative events can be conveyed with pictures and words

A central characteristic of visual narratives is that information is presented in different codalities (e.g., as pictures or a text; for an example see Fig. 1). Whereas in the example comic strip in Fig. 1A all relevant information is pictorially depicted, the critical information of the third panel was replaced with textual information in Fig. 1B. To comprehend the fourth panel of this comic strip, it is essential to process and relate both the pictorial and the textual information of the clip. The present study examined how switching between the codality of narrative information affects narrative comprehension. More specifically, we studied *narrative comprehension processes* using visual narratives and manipulated whether specific information is conveyed pictorially or textually, thus addressing the question of how information from different codalities is integrated into a coherent mental representation of the narration.

In the present project, we use the term *codality*, following the distinction between the *modality* and codality of information (Clark & Salomon, 1986). While the modality refers to the sensory channel through which the information to be conveyed is perceptually processed (e.g., visual or auditory), the codality refers to the specific information coding (e.g., verbal, pictorial, symbolic). For instance, an “apple” can be represented in a photograph (visual modality, pictorial code), be written (visual modality, verbal code), or be represented by an icon (visual modality, symbolic code). Thus, a picture story with isolated text elements is *uni-modal* (visual) but *multi-codal* (pictorial and verbal). This definition differs from those used in recent studies in which a picture story with isolated text elements was labelled “uni-sensory cross-modal” (Manfredi, Cohn, & Kutas, 2017).

### Understanding multi-codal narratives: The general comprehension skill

The starting point of our study was the seminal work of Gernsbacher and colleagues who argued that there exists a *general comprehension skill* (Gernsbacher, Varner, & Faust, 1990). The ability to understand verbal, text-based stories is strongly correlated with the ability to understand non-verbal, picture stories. The associated cognitive processes and mechanisms of the general comprehension skill are also reflected in the *structure building framework* (Gernsbacher, 1997), a system that uses the following processes to form a cohesive mental representation or structure of a story. In the first step of building up such a structure, a foundation is laid. A coherent mental representation is then developed by *mapping* new information to this foundation. If, however, the new incoming information cannot be mapped onto the current structure, comprehenders lay a new foundation (*shifting*) to build up a new structure (Gernsbacher et al., 1990). Experimental evidence for the existence of these processes comes from viewing-time studies, which found longer viewing times for the first sentence or picture of a story (*shifting and laying a foundation*) as compared to the viewing times observed for subsequent sentences or pictures (*mapping*) (Cohn & Wittenberg, 2015; Haberlandt, 1984; Hard, Recchia, & Tversky, 2011).

### Representing dynamic events in situation models

Research on situation models – mental representations of the current state of a narration in working memory – also refers to the processes proposed in Gernsbacher’s *structure building framework* by proposing that the processes involved in building up a situation model (i.e., mental representations of certain events) are independent of stimulus codality (Zwaan & Radvansky, 1998). The underlying basis of all these processes might be amodal processing of information. Van Dijk and Kintsch (1983) argue that reading a text does not only create a mental representation of the text itself but also a semantic representation and a *mental representation of the situation* described in the text. According to Zwaan and Radvansky, such a situation model, which was originally only used to explain word processing, should therefore be accessible to various codalities. Although most research on this event-indexing model and the construction of mental models during narrative comprehension is based on text-based stimulus material (Radvansky & Copeland, 2010), there is converging evidence that comprehenders also construct and update mental models while watching silent movies (Magliano & Zacks, 2011) and audiovisual narratives (Huff, Meitz, & Papenmeier, 2014) or listening to audio dramas (Huff et al., 2018; Papenmeier, Maurer, & Huff, 2019). This suggests that basic processes of narrative understanding are independent of codality (Meitz, Meyerhoff, & Huff, 2019).

### Evidence for comparable narrative comprehension processes across codalities

Studies on event segmentation also point to similarities between different codalities (Baggett, 1979; Magliano, Kopp, McNerney, Radvansky, & Zacks, 2012). Event segmentation theory (Zacks, Speer, Swallow, Braver, & Reynolds, 2007) proposes that comprehenders perceive dynamic events by segmenting them into distinct units (Newtson, 1976). According to event segmentation theory, event models (which are similar to situation models) guide comprehenders’ perceptual predictions, which are the basis of dynamic event perception. If the perceptual predictions do not correspond to reality, the event models are updated, and an event boundary is perceived (Zacks et al., 2007). Importantly, the basic assumptions of the event segmentation theory are independent of stimulus codality. Baggett (1979) had already shown that participants segmented a narrative into similar episodes regardless of the codality of presentation (i.e., perceived the same structure). Further, Magliano et al. (2012) also found that subjects perceived similar event boundaries, regardless of whether they read a narrative as text or saw it as an image story. These results suggest that the event boundaries are perceived independently of stimulus codality (Zacks et al., 2007).

Taken together, there is evidence that the basic processes of constructing and updating event models are independent of stimulus codality. In addition, information represented in situation models are the basis for inference generation processes. Such processes help in coping with missing or incomplete information. The generation of bridging inferences requires resources (Fincher-Kiefer & D’Agostino, 2004; Magliano, Kopp, Higgs, & Rapp, 2017; Magliano, Larson, Higgs, & Loschky, 2016). If, for example, the third panel (“Boy pours a bucket of water through the window”) in the comic depicted in Fig. 1A was omitted, this missing information would have to be generated while comprehending the fourth panel (“The wet dad looks out of the window, grumbling”). The viewing time paradigm allows us to measure inference generation processes (H. H. Clark, 1977; Graesser, Singer, & Trabasso, 1994). Here, participants are presented with one panel of a comic after the other. They are asked to focus on comprehending the story and to press a dedicated button to go to the next panel. The time participants spend processing a panel before proceeding to the next panel is the viewing time. By measuring the viewing times while comprehending picture stories, Magliano et al. (2016) have demonstrated that inference generation requires more resources if an image is not presented. Using the dual-task paradigm, this study further showed that both visuospatial and linguistic working memory processes support inference generation. However, as this study only used visual material – the picture stories from the *Boy, Dog, Frog* series (Mayer, 1967, 1969, 1973, 1974; Mayer & Mayer, 1971, 1975) – it remains an open question how information of different codalities (i.e., pictures *and* text) is integrated during narrative comprehension.

### Integrating text and pictures during narrative understanding

Manfredi et al. (2017) conducted a first study addressing this topic using event-related brain potential (ERP) measures by investigating how different forms of text-based bridging-event information are integrated. The authors replaced individual images of a picture story with lexically diverse words and examined the effects on understanding. Descriptive words (e.g., *Impact!*) were found to be more unexpected than onomatopoeic words (e.g., *Bang!*). Accordingly, Manfredi et al. explained their results with the higher probability of the occurrence of onomatopoeic words in comics, as a recent corpus analysis has shown (Pratha, Avunjian, & Cohn, 2016). Importantly, although the focus of the Manfredi et al. (2017) study is on integration processes of information of different codalities, there was no baseline condition with just visual information. Thus, it is an open empirical question how the studied text-based information relates to a visual version of it.

Text-based and pictorial information differ in various ways. Text-based narrative techniques (e.g., indirect speech or an omniscient narrator) can be used to describe a protagonist’s *internal state* such as emotions, goals, intentions, or knowledge (Magliano, Loschky, Clinton, & Larson, 2013). Depicting such internal states in the context of picture stories and comics is more subtle and is often realized implicitly via the protagonist’s posture or mimic (Magliano et al., 2012, 2013). Empirical evidence for such differences comes from studies directly comparing pictorial and textual information processing. An important difference is that, when reading text and looking at picture stories, different areas of the brain are involved (Ganis, Kutas, & Sereno, 1996; Magliano et al., 2013). This is also reflected in the dual-coding theory (Paivio & Csapo, 1969) proposing two functional independent but interrelated cognitive subsystems. Thus, it seems reasonable to assume that explicit and implicit depictions of internal states entail different processing strategies during cross-codal information processing. This is also true for the processing of spatial relations. It was shown that reading a text describing the spatial locations of objects within a building (Morrow, Greenspan, & Bower, 1987) and navigating within a (pictorial) virtual environment (Tamplin, Krawietz, Radvansky, & Copeland, 2013) resulted in profound differences in memory performance. In particular, whereas reading led to the effect that the accessibility of information about read objects decreased with increasing distance, navigating through the same virtual environment showed no spatial gradient for object memory. Instead, object memory was highest for objects in the same (location) room but lower for objects in previously visited rooms (Tamplin et al., 2013).

Taken together, although narrative understanding is assumed to be a process that is independent of stimulus codality (Gernsbacher et al., 1990), there are also reasons to consider that each codality (i.e., text-based, pictorial) entails different processing strategies (e.g., Tamplin et al. 2013) that might also influence cross-codal integration in narrative understanding. Apart from a notable exception (Manfredi et al., 2017), there is no study on cross-codal integration effects in the domain of narrative understanding.

## Experimental overview and hypotheses

We report three experiments that tested cross-codal integration of bridging-event information during narrative understanding. The central manipulation across all experiments was the codality of bridging-event information. In all three experiments, we instructed the participants to focus on comprehending the depicted narrative and reinforced this instruction by asking them to write a short summary after each narrative. Because this was done to motivate the participants to comprehend the stories, the summary protocols were not analyzed. We measured the participants’ viewing times as the dependent variable for invested mental effort. If the processing of bridging-event information is independent of its codality (i.e., pictorial, textual), we expect no difference between viewing times for the information that follows the pictorial or textual bridging-event information. However, if codality matters, there should be a significant increase in viewing times after the textual bridging-event information. This would reflect costs related with integrating cross-codal information into the mental representation of the narration (i.e., code switching).

**Fig. 2:**
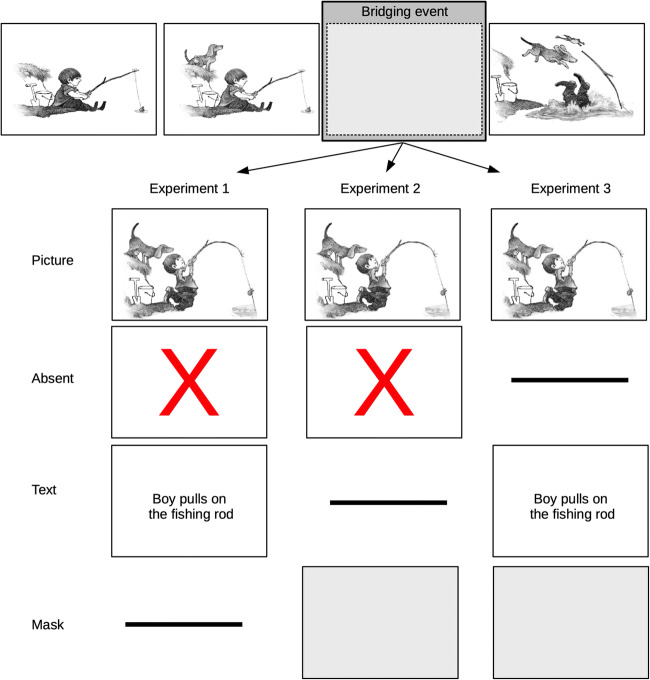
Schematic depiction of the design and material of a target episode used in this study. We tested three conditions in each experiment (Experiment 1: absent, picture, text; Experiment 2: absent, picture, blank; Experiment 3: text, picture, blank). The horizontal line indicates that this condition was not realized in the respective experiment. Please note that the *text* was presented in German

In each experiment, the participants saw 18 target episodes distributed across six visual narratives. Each target episode consisted of a *beginning state*, a *bridging event*, an *end state*, and an *end state + 1*. In Experiment 1, *bridging-event information* was either present (as picture or text; a pilot study confirmed that both conditions were not different with regard to information richness) or absent (i.e., not shown, see Fig. 2). We expected the viewing times for the end-state picture to be higher in the absent condition than in the picture condition (replicating Magliano et al., 2016). Further, if the processing of bridging-event information is independent of codality (Magliano et al., 2012), there should be no differences between the text and picture condition. If, however, this process is codality dependent, the viewing times for the end-state pictures should be higher in the text as compared to the picture conditions. To anticipate our results, we observed a codality effect – the viewing times in the text condition were longer than in the picture condition and comparable to those in the absent condition. We propose, that this effect might be traced back to the recoding inference (Adaval & Wyer Jr., 2004) that is observed when the codality of new information is different from the codality of already encoded information. In Experiment 2, we replaced the bridging-event information with a blank panel to study how distorting narrative continuity influences the narrative processing of *end-state picture*s (Cohn & Wittenberg, 2015). *Bridging event information* was either present (as picture) or absent (not shown or blank panel, see Fig. 2). As in Experiment 1, we expected viewing times in the absent condition to be higher compared to the picture condition. If distorting narrative continuity with a blank panel has effects above the regular integration cost, the viewing times should be higher than in the absent/not shown-condition. Thus, Experiment 2 establishes a baseline for integration costs. Finally, in Experiment 3, we contrasted viewing times in the text and blank condition. Bridging event information was *present* (as picture or text) or *replaced with a blank panel*. As in Experiments 1 and 2, we expected the viewing times in the text condition to be higher than in the picture condition but shorter than in the blank-panel condition.

## Experiment 1 – Codality

In Experiment 1, *bridging-event information* was present (as picture or text; a pilot study confirmed that the two conditions were not different with regard to information richness) or absent (i.e., not shown, Fig. 2). Replicating Magliano et al. (2016), we expected the viewing times for the end-state picture to be higher in the absent condition than in the picture condition. Further, if the processing of bridging-event information is independent of codality (Magliano et al., 2012), there should be no differences between the text and the picture condition. If, however, this process is codality dependent, the viewing times for the end-state pictures should be higher in the text as compared to the picture conditions.

### Participants

Eighty-two students (66 female, 16 male, age 19–42 years, *M* = 23.55 years) from the University of Tübingen participated in exchange for course credit or monetary compensation of 8€.

### Material

Similar to Magliano et al. (2016), we used six picture stories from the *Boy, Dog, Frog* series (Mayer, 1967, 1969, 1973, 1974; Mayer & Mayer, 1971, 1975) as stimulus material. Each narrative included 24–26 single pictures. We used the same edited pictures (e.g., with removed background information) as Magliano et al. (2016).^1^ In contrast to the latter study, which comprised 24 target episodes, we chose to only use those target episodes (beginning state, bridging state, end state, end state + 1) that did not directly connect with other critical events of the story (such as other target episodes or the end of the story). With this selection we ensured that there was at least one “neutral” panel – that was neither an end *state + 1* panel nor a *beginning state* – between two episodes. This resulted in 18 target episodes across all stories (three per story: absent, picture, and text). Each target episode consisted of a *beginning state*, a *bridging event*, an *end state*, and an *end state + 1*. Magliano et al. (2016) have validated the existence of bridging events (i.e., they showed that participants mention the bridging-event information more often in the absent than in the present condition because inferring an action typically results in a higher activated representation than simply seeing an action). Pictures had a resolution of 1,280 × 720 pixels and were presented full-screen.

In Experiment 1, the bridging event was presented as a picture or as a text or was absent. A pilot study confirmed that there is a close match between pictures and corresponding texts.

### Pilot study

**Fig. 3:**
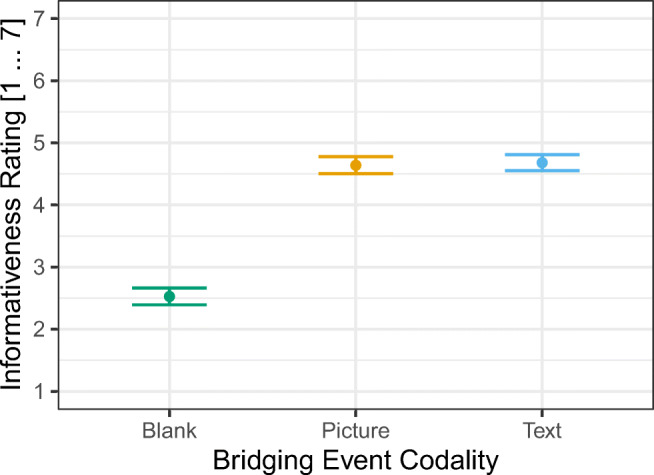
Mean informativeness rating for the different bridging-event information codalities. Error bars represent the standard error of the mean

**Fig. 4:**
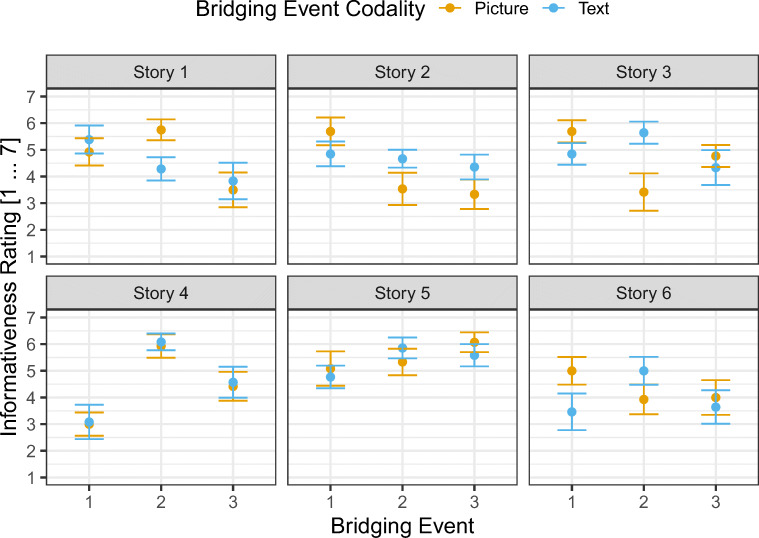
Mean informativeness ratings as a function of story, bridging event, and codality (picture, text) separately for each item. Error bars represent the standard error of the mean

We designed the text versions in close orientation to Magliano et al. (2012) such that the text conveys the central meaning of the corresponding picture within its context (see Fig. 2 for an example). In a *pilot study,* we presented *N* = 42 participants with the bridging events of all 18 target episodes. The critical information was presented as picture, text, or replaced with a blank panel (see Fig. 2). We counterbalanced this factor across participants. Participants saw all four panels of the target episodes on-screen. Each panel was faded in after a delay of 2 s to its predecessor beginning with the left-most picture. After the final information was faded in, a rating scale appeared asking the participants “how informative is the information presented on the third panel for the understanding of the pictorial information of the final panel?” They were asked to make their judgment on a 7-point scale by mouse-click (1 = "very bad", ... 7 = "very good"). We excluded three participants from the analysis because there were no responses recorded for these participants. We aggregated the data on the participant level and submitted these data to a one-way ANOVA with bridging event codality as within-subjects factor (Blank, Picture, Text), *F*(2, 76) = 34.40, *p* < .001 (see Fig. 3). Holm-Bonferroni corrected pairwise t-tests confirmed that the informativeness rating for the Picture and Text condition did not differ (*p* = .813) but are both higher (i.e., more informative for the understanding of the final pictorial panel) than the Blank condition (both *p*s < .001). As can be seen in Fig. 4, the informativeness ratings for all bridging events are sufficiently similar across codalities. Thus, we are confident that the text items adequately describe the narrative.

### Design

Experiment 1 employed a 3 (bridging event: absent, picture, text) × 2 (image sequence: end state, end state + 1) within-participants design. As the dependent measure, we used the viewing times spent on the respective images. In order to eliminate any potential influence from the serial order of the presentation of the codality conditions within a story (Absent, Picture, Text), we used a completely balanced Latin-square design resulting in six counterbalancing conditions (see Appendix Table 2). The serial order of the story presentation within each counterbalancing condition was randomized across participants. Randomization of story order was done after counterbalancing. We tested at least 13 and a maximum of 14 participants per counterbalancing condition (see Appendix Table 2).

### Procedure

After providing informed consent, the participants were instructed that they would see six picture stories in which some information is provided as text. The instructions included an example demonstrating the central manipulations of this experiment (a cartoon depicting a mouse offering a bouquet of flowers to an elephant and the corresponding textual description thereof). We further instructed them to focus on comprehending the stories and reinforced this instruction by asking them to write a short summary at the end of each story. The experiment was programmed in *PsychoPy* (Peirce, 2007; Peirce et al., 2019) and employed a standard viewing time procedure, which is similar to the reading time paradigm (Zwaan, Magliano, & Graesser, 1995b). Each picture of a story was presented separately on-screen, and the participants pressed the spacebar on a standard keyboard to view the subsequent picture in a self-paced manner. Viewing time was defined as the time between two button presses. Between the stories, the participants were allowed to take a short break. They proceeded to the next story by clicking the *w*-key on the keyboard. The experiment lasted approximately 1 h.

### Results

Data cleaning was similar to Magliano et al. (2016). First, the data were trimmed using the following criterion-based trimming rule, which considers mean fixation duration of scene viewing and the duration of a simple reaction. Based on this, the shortest adequate viewing time was set to 0.48 s (26 of 3,936 trials – 0.66% – were excluded based on this criterion), the longest adequate viewing time was set to 20 s (38 of 3,936 trials – 0.97% – were excluded based on this criterion). Second, viewing times larger than 3 standard deviations above the arithmetical mean (after criterion trimming) for each of the experimental conditions were removed. Based on this normative trimming, we removed 89 (2.26%) trials.

**Fig. 5:**
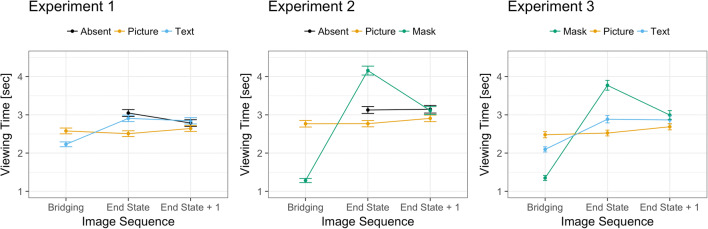
Mean viewing times (in seconds) for Experiments 1, 2, and 3 as a function of condition (black: absent; orange: picture; blue: text; green: blank) and image sequence. Error bars represent the standard error of the mean (*SEM*). *Note:* In the analyses, we only included the *end state* and *end state + 1* pictures

We analyzed the log-transformed viewing-time data using linear mixed-effects models using the *lme4 package* (Bates et al. 2014) with maximal random effects structure, including condition (absent, picture, text) and image sequence (end state, end state + 1) and their interactions as fixed effects, and random slopes for both participants and story (Barr, Levy, Scheepers, & Tily, 2013). We analyzed the model’s parameters with a type-II ANOVA, using the ANOVA function of the car package (Fox & Weisberg, 2010). As expected, the results showed a significant interaction of condition and image sequence, χ^2^(2) = 11.91, *p* = .003 (see Fig. 5). For the end-state picture, viewing times were longer in the absent and text conditions as compared to the picture condition; there was no difference in viewing times for the *end-state picture* in the absent and text conditions. We did not observe a difference between the experimental conditions for the *end state + 1* pictures (see Table 1 for details), indicating that cross-codal integration does not extend beyond the *end-state picture*s. Further, the main effect for condition was significant, χ^2^(2) = 28.88, *p* < .001; the main effect for image sequence was not, χ^2^(1) = 1.51, *p* = .218.

**Table 1:** Differences of least squares means for the interaction effects of Experiments 1, 2, and 3. Tukey adjusted t-tests

Experiment	Image sequence	Condition	Estimate	*SE*	*df*	*t*	*p*
Experiment 1	End state	Absent – Picture	0.2	0.04	108.9	5.33	< .001
		Absent – Text	0.0	0.04	19.3	0.52	.611
		Picture – Text	-0.2	0.03	41.2	-4.88	< .001
	End state + 1	Absent – Picture	0.0	0.04	109.0	0.85	.397
		Absent – Text	0.0	0.04	19.0	-1.02	.319
		Picture – Text	-0.1	0.03	40.7	-2.00	.052
Experiment 2	End state	Absent – Picture	0.1	0.03	444.0	4.10	< .001
		Absent – Blank	-0.3	0.04	342.2	-7.75	< .001
		Picture – Blank	-0.4	0.04	205.4	-11.84	< .001
	End state + 1	Absent – Picture	0.1	0.04	444.0	1.88	.061
		Absent – Blank	0.0	0.03	332.7	0.59	.558
		Picture – Blank	0.0	0.04	208.5	-1.28	.201
Experiment 3	End state	Blank – Picture	0.4	0.05	19.8	8.09	< .001
		Blank – Text	0.3	0.05	39.1	6.15	< .001
		Picture – Text	-0.1	0.04	91.5	-2.91	.004
	End state + 1	Blank – Picture	0.1	0.05	20.3	1.88	.074
		Blank – Text	0.1	0.05	39.7	1.50	.142
		Picture – Text	0.0	0.04	96.7	-0.59	.555

### Discussion

Experiment 1 showed how the codality of bridging-event information influences narrative comprehension. Conceptually replicating Experiment 1 by Magliano et al. (2016), we added a third condition in which bridging-event information was presented as text. Using the viewing time paradigm and well-documented stimulus material, we could show that text-based bridging-event information is harder to integrate than pictorial bridging-event information. This suggests that narrative understanding is codality dependent.

We propose that the reported codality effect might result from interference of pictorial and text-based information. Literature on the verbal overshadowing effect (Alogna et al., 2014; Schooler & Engstler-Schooler, 1990) reports lower visual recognition performance after participants have verbally described or read a description of a previously seen visual stimulus (such as a face) as compared to a control condition without verbal descriptions (Dodson, Johnson, & Schooler, 1997; Huff & Schwan, 2008). One explanation for verbal overshadowing refers to recoding-interference (Adaval & Wyer Jr., 2004), which can be observed if the codality of new information is different from the codality of already encoded information. Applied to the results of the present experiment, the text-based bridging-event information might need to be re-coded in order to be integrated into the mental representation that is based on the picture-based information of the visual narrative. This recoding-interference effect is reflected by longer viewing times of the end-state picture.

Viewing times for the end-state picture in the text condition were not different from those in the absent condition. Assuming that generating bridging inferences in the absent condition does not trigger a reset of the present situation model (Zwaan & Radvansky, 1998), we conclude that this is also not the case in the text condition. Instead, we propose that the present situation model is preserved and that the increased viewing times in the text condition reflect cross-codal integration costs resulting from recoding-interference.

To assess the relative costs of integrating cross-codal information, we conducted two further experiments. Experiment 2 establishes a baseline for integration costs by introducing a condition that is known to elicit long viewing times. More specifically, we replaced the bridging-event information with a blank panel. Finally, Experiment 3 directly compares the text and picture conditions with the blank panel (baseline) condition.

## Experiment 2 – blank panel

In Experiment 2, we introduce a condition that is known to cause severe violations of narrative continuity, thus providing an estimate of the baseline for integration costs. Recent research has shown that replacing the original contents of a panel with a white panel results in increased viewing times for the subsequent image (Cohn & Wittenberg, 2015). According to the authors, semantically impoverished panels require extensive inference generation processes. However, unlike Magliano et al. (2016), the Cohn and Wittenberg (2015) study did not include an absent condition. It is thus an open empirical question how the absent condition compares to the blank-panel conditions. To link the results of Experiment 1 – increased viewing times in the absent and text conditions – with the existing literature, we tested how replacing bridging-event information with a blank panel compares to the absent condition.

Bridging-event information was either presented (as picture) or not presented (absent vs. blank panel, see Fig. 2). Viewing times in conditions not presenting bridging-event information should be higher than in a condition presenting this information. Further, a blank panel should elicit longer viewing times spent on the end-state picture as compared to the absent condition.

### Participants

Eighty students (59 female, 21 male, age 18–37 years, *M* = 23.60 years) from the University of Tübingen and the University of Jena participated in exchange for course credit or monetary compensation of 8€. Due to computer failure, we lost data of three participants. Thus, the final sample consisted of 77 participants.

### Stimulus material, design, and procedure

Stimulus material, design, and procedure were identical to Experiment 1 with the exception that we presented a blank panel instead of the respective text (see Fig. 2). As the blank panel, we presented a light gray-white pattern. The blank panel was presented in full screen, like the pictures. As in Experiment 1, there were six counterbalancing conditions. We tested at least 12 and a maximum of 14 participants per counterbalancing condition.

### Results

Data cleaning was similar to Experiment 1. Again, the shortest adequate viewing time was set to 0.48 s (21 of 3,696 trials – 0.55% – were excluded based on this criterion), the longest adequate viewing time was set to 20 s (34 of 3,696 trials – 0.89% – were excluded based on this criterion). Second, viewing times larger than 3 standard deviations above the arithmetical mean for each of the experimental conditions were removed. Based on this normative trimming, we removed 76 (1.98%) trials.

The statistical analysis was similar to Experiment 1. The results showed a significant interaction of condition and image sequence, χ^2^(2) = 62.92, *p* < .001 (see Fig. 5). For the end-state picture, the viewing times were longer in the blank-panel condition than in the absent and picture conditions. Further, the viewing times were longer in the absent than in the picture condition, thus replicating Experiment 1. As in Experiment 1, we did not observe a difference between the experimental condition for the end state + 1 pictures, again showing that integration costs are restricted to the *end-state picture* (see Table 1 for details). In addition, the main effects for condition, χ^2^(2) = 84.47, *p* < .001, and image sequence, χ^2^(1) = 23.63, *p* < .001, were significant.

### Discussion

Experiment 2 provided us with a baseline of integration costs. Compared to the absent condition, replacing bridging-event information with a blank panel significantly degraded narrative continuity as the prolonged viewing times showed. Assuming that participants are able to integrate information before and after the blank panel into a coherent mental representation of the narrative, we presume that this is even more true for the absent condition.

In Experiment 2, we closed the gap in existing research by manipulating whether bridging-event information was presented as picture, absent, or replaced with a blank panel. The results confirmed our expectations. Longer viewing times in the blank-panel condition as compared to the absent condition suggest that elaboration in the blank-panel condition is higher than in the absent condition.

To investigate the costs of integrating information of different codalities, we directly compared the text and picture conditions of Experiment 1 with the blank-panel conditions of Experiment 2.

## Experiment 3 – Codality versus blank panel

In Experiment 3, we tested whether replacing bridging-event information with a blank panel increases the viewing times more than the text-based bridging events used in Experiment 1. Bridging-event information was either present (as picture or text) or absent (blank panel, see Fig. 2). A blank panel should elicit longer viewing times for the end-state picture as compared to the text and picture conditions. Further, we expected longer viewing times for the end-state picture in the text as compared to the picture condition (replication of Experiment 1).

### Participants

Seventy-six students (48 female, 28 male, age 19–67 years, *M* = 27.42 years) from the University of Tübingen and University of Jena participated in exchange for course credit or monetary compensation of 8€.

### Stimulus material, design, and procedure

Stimulus material, design, and procedure were identical to Experiment 1 with the exception that we directly compared the three-condition picture and text of Experiment 1 and blank panel of Experiment 2 (see Fig. 2). As in Experiment 1, there were six counterbalancing conditions. We tested at least nine and a maximum of 14 participants per counterbalancing condition.

### Results

Data cleaning was similar to Experiment 1. Again, the shortest adequate viewing time was set to 0.48 s (38 of 3,838 trials – 0.99% – were excluded based on this criterion), the longest adequate viewing time was set to 20 s (65 of 3,838 trials – 1.69% – were excluded based on this criterion). Second, viewing times larger than 3 standard deviations above the arithmetical mean for each of the experimental conditions were removed. Based on this normative trimming, we removed 85 (2.21%) trials.

The statistical analysis was similar to Experiment 1. The results showed a significant interaction of condition and image sequence, χ^2^(2) = 33.64, *p* < .001 (see Fig. 5, right panel). For the end-state picture, the viewing times were longest in the blank-panel condition. Further, the viewing times in the text condition were longer as compared to the picture condition, thus replicating Experiment 1. Again, we did not observe a difference between experimental conditions for the end state + 1 pictures (see Table 1 for details). In addition, the main effects for condition, χ^2^(2) = 35.49, *p* < .001, and image sequence, χ^2^(1) = 7.40, *p* = .007, were significant.

### Discussion

The results of Experiment 3 showed that the distortion caused by replacing the visual content of a panel with a blank panel is larger than the distortion caused by cross-codal integration processes. We thus conclude that cross-codal integration is possible but requires extra elaboration in contrast to the purely pictorial presentation; however, it is less effortful than providing a blank panel that does not constrain learners’ narrative comprehension.

## General discussion

Narrative comprehension is based on the online generation of inferences between explicitly depicted parts of the unfolding event. In the present study, we tested whether bridging-event information processing is codality dependent. In particular, we studied if and how text-based bridging-event information is integrated in the mental representation of a visual narrative. This allows us to directly assess cross-codal integration costs. Importantly, the mental representation of bridging-event information can be based on pictorial or textual information, or on information that is generated by inferences.

In Experiment 1, we showed that there are cross-codal integration costs. We speculate that these costs might be traced back to recoding interference effects. Recoding interference effects can be observed if the codality of new incoming information is different from the codality of already existing information in working memory (Huff & Schwan, 2008). Integrating both types of information depends on the recoding of the new information, which requires mental effort. In Experiment 2, we introduced a baseline of integration costs by adding a condition in which we replaced the bridging-event information with a blank panel (Cohn & Wittenberg, 2015). As the viewing times in the absent condition were far below the viewing times in the blank-panel condition (i.e. baseline), we conclude that generating bridging event inferences is possible in the absent condition. In Experiment 3, we directly compared the integration of text- and picture-based bridging-event information with a condition in which this information was replaced with a blank panel. Because viewing times in the text and picture condition were significantly lower than in the blank-panel condition, we conclude that integration is possible but associated with costs that might be traced back to recoding interference effects (Adaval & Wyer Jr., 2004; Huff & Schwan, 2008).

The present results suggest recoding interference effects as a result of cross-codal information presentation. This might be traced back to the capacity of pictorial and text information to convey implicit information. For example, whereas internal states of a character (such as *joy* or *grief*) can be described explicitly using text information, pictorial code is implicit – the comprehender must extract this information from the facial expressions of the characters.

### Theoretical implications

Theories on narrative understanding have not looked very much into the cost of switching between different codalities until now. On the contrary, the structure-building framework (Gernsbacher et al., 1990), event indexing (Zwaan, Langston, & Graesser, 1995a), as well as theories focusing on the perception and comprehension of natural dynamic events (i.e., complex actions) such as the event segmentation theory (Zacks et al., 2007) make no explicit predictions about the processing of cross-codal information. Instead, research until now has mainly focused on uni-codal processing and – as the observed effects were comparable across codalities (e.g., with regard to memory for visual and auditory events) – has speculated that information processing might be independent of stimulus codality. The present experiments have shown that the integration of information from different codalities is possible but requires extra resources. In the following, we outline how the identified processes help to understand the basic processes of initiating and updating working memory representations of a narration or depicted plot (i.e., situation models, event models) and how existing theories need to be updated to take cross-codal information processing into consideration.

A first important observation is that theories addressing dynamic information processing are *uni-codal* (but are often used to also explain multi-codal phenomena); that is, they begin with a specific observation and demonstrate the central principles of the respective theory using *uni-codal* stimulus material. For example, theoretical models of discourse processing use texts (Kintsch & Van Dijk, 1978), whereas theories on event cognition use video clips without sound information as the stimulus material (Zacks, Kurby, Eisenberg, & Haroutunian, 2011). In a next step, research on these theories often extend their scope by using stimulus material of a different codality to show that the basic principles also apply. Perhaps the most prominent example is the study that identified the general comprehension skill (Gernsbacher et al., 1990) in which the authors compared the understanding of auditory, text, and picture stories and found high correlations across the different codalities. Further, Magliano and colleagues compared event segmentation behavior for the pictorial and a text version of the *Boy, Dog, Frog* stories, which was remarkably comparable across codalities (Magliano et al., 2012). Eventually, some research then used multi-codal stimulus material (such as films with sound information) to study the related principles further (Huff et al., 2014; Meitz et al., 2019).

All of this research shares the fact that information codality is *constant during presentation* (i.e., texts, pictures, silent movies, films with sound). According to Van Dijk and Kintsch (1983), a situation model arises as the result of processing the text information. Presenting cross-codal information for the first time (such as a text in a comic) now initiates a first mental representation of the respective codality (i.e., text base). Research on multimedia learning has addressed the issue of learners having to handle more than one codality at a time and has proposed that there is a pictorial base as a counterpart of the text base in discourse processing (Mayer, 1997; Schnotz & Bannert, 2003). Recoding interference effects as observed in this study now might be traced back to integrating the information from the text base into the mental representation that was originally encoded from picture-based information.

The present results are consistent with a very recent theoretical approach – Scene Perception and Event Comprehension Theory (SPECT) – addressing the interplay of frontend and backend processes during narrative understanding (Loschky, Larson, Smith, & Magliano, 2019). Whereas frontend processing is related to information extraction (that can be measured using eye-movements), backend processing is related to memory (such as working memory and long-term memory). Although this SPECT theory is silent with regard to codality switching and integration, it is reasonable to assume that the integration costs observed in the present set of experiments can be traced back to encoding – that is frontend processing. Studying these integration costs using eye-tracking methods seems to be an auspicious approach as eye-tracking studies have shown that comic readers spend more time on panels also including texts as compared to panels with just pictorial content (Chiba, Tanaka, Shoji, & Toyama, 2007; Laubrock, Hohenstein, & Kümmerer, 2018). Such a study could also address the alternative explanation of the present findings according to which codality switching just surprises participants. In this case, increased viewing times would not reflect integration but rather surprise due to the presentation of a panel in an unexpected codality (Pettijohn & Radvansky, 2016). Although we consider this explanation unlikely because participants were completely informed about all experimental conditions, a study addressing this topic could compare viewing times for text information that is compatible with the narrative with text information that is incompatible with the narrative. If it is surprise alone, we expect no viewing time difference between these two conditions. If, however, expectancy plays a role, viewing times for the incompatible text-information should be higher. Further, eye-movement patterns could also help to identify processing that is related to surprise (Blanchard & Iran-Nejad, 1987).

Taken together, we propose that the theoretical models addressing the question of how comprehenders integrate dynamic information into a coherent mental representation of the unfolding event (including event indexing, the construction integration model, and SPECT) should consider information codality.

### Towards a model of cross-codal event comprehension

The present set of experiments suggest that switching codalites during narrative understanding causes costs that might be traced back to recoding interference. In the following, we outline a model focusing on cross-codal information processing in narrative understanding and explaining the origins of recoding interference.

We refer to the idea of codality-specific bases (i.e., a text-base and a picture-base) for initial processing from models explaining learning in multimedia environments (Schnotz & Bannert, 2003) and to the idea of perceptual simulation of verbally described events from research on embodied cognition (e.g., Zwaan, 2016). A critical question with regard to cross-codal event comprehension and the emergence of recoding interference, respectively, refers to the time-course of information processing. As baseline, we consider narrations consisting of uni-codal information (such as picture stories or textual narrations). Since no code-switching is necessary here, we will assume that uni-codal information is processed consecutively and integrated into the event model immediately during reception. For narrations consisting of multi-codal information, code-switching is essential for successful event comprehension. Incoming information is first represented in codality-specific bases (i.e., a text-base and a picture-base) in which initial processing takes place and from which the selected information is then transferred and integrated into a mental representation. According to Schnotz and Bannert (2003), a central prerequisite for successful integration of information from different codality-specific bases into a coherent mental representation is the simultaneous presence of corresponding information in working memory and the subsequent mapping of this information. Yet, event comprehension differs from multimedia learning in the way stimulus material is presented. Whereas the learning materials in the Schnotz studies were all presented simultaneously to the learners, the experimental materials that are used to investigate narrative comprehension of events usually unfold over time, so that not all of the information is available at the same time. That is, in order to build up a comprehensive event model representing the narrative plot, comprehenders have to hold the already presented but no longer available information in working memory. We propose that perceptual simulation (e.g., Barsalou, 1999, 2008; Zwaan, 2016) might be the underlying mechanism that prepares the text-based information for integration into the event model of the narrative plot.

Two processing- and integration-scenarios for multi-codal event comprehension are possible. They differ with respect to the time point of integration. The immediate-integration scenario assumes that the information of the different, second codality is integrated during reception and code-switching. This requires additional mental effort for code switching at the very time-point the other codality in presented. This is the point when recoding interference emerges. The late-integration scenario assumes that information from the different codality (i.e., the text panel in the present experiments) is not immediately integrated within the mental representation of the narration. Rather, this scenario assumes that integration – and recoding interference, respectively – occur at the time point comprehenders realize that there is missing information for understanding the plot. In this case, information from the other codality specific base (i.e., the text base) is used to complement the mental representation of the narration. That is, the cost of integrating cross-codal information occurs not at the time point of initial processing (i.e., reading the text panel) but rather at a later time point at which the missing information is integrated into the mental representation.

To test the two different scenarios, we propose an experiment that directly compares a picture-text condition – a visual narrative with the bridging event replaced with equivalent textual information (such as the text condition of the present set of experiments) – with a text-only condition – a narrative consisting of text panels only. In such an experiment, viewing times for the bridging event and the end-state panels should be compared. For immediate-integration, we would expect a significant viewing time difference at the bridging event panel with longer viewing times for the picture-text as compared to the text-only condition. In this case, the prolonged viewing times observed in the picture-text condition at the end-state panel would reflect the costs related with switching back to the visual codality. If, however, integration occurs when noticing the missing information (i.e., at the end-state panel), we expect no difference in viewing times between those two conditions at the bridging event panel. Instead, the prolonged viewing times in the picture-text condition at the end-state panel would reflect costs associated with integration (i.e., recoding interference).

### Limitations and further research

In the present experiments, we studied cross-codal effects in narrative comprehension. We propose that cross-codal integration is possible but associated with integration costs. Yet, we only tested how text-based information is integrated when comprehending visual narratives. Future research needs to address the question of how pictorial information is integrated when comprehending text-based narratives. This integrates nicely with a recent corpus analysis studying American superhero comics between the 1940s and 2010s by Cohn, Taylor, and Pederson (2017). More specifically, this study examined the development of interactions between textual and pictorial components of comics across six centuries. The results have shown that there was a shift in storytelling towards the pictorial elements of the comics across the years. Thus, a potential future study testing the boundaries of the reported effect could use comics from different centuries studying cross-codal integration processes.

With the present set of experiments, we studied cross-codal integration in visual narratives. This complex set of interaction effects could also have been studied in a single experiment with the conditions text, picture, absent, and blank panel. Using our stimulus material would have thus resulted in a lower number of repeated measurements per condition and to less accurate estimates as a consequence.

To keep participants’ attention on understanding the visual narratives, we asked them to write a short summary after each comic. This is quite common in research using visual narratives as stimulus material (e.g., Magliano et al., 2016). Whereas this instruction may result in increased attention and thus result in more elaborate processing than naturalistic scenarios of processing visual narratives, we know of no study that has directly tested the effect of this instruction on participants’ processing and understanding of comics. Further research is necessary to address this research question.

### Conclusion

Narrative comprehension often requires the processing of information that is provided in different codalities (such as pictures and text). This information has to be integrated during comprehension. The present set of experiments has demonstrated that processing text-based bridging-event information requires more time than processing equivalent picture-based bridging-event information. Because replacing the critical information with a blank panel entails even more time for processing, we have proposed that information depicted in a different codality is used for comprehension. Integrating the text-based information into the mental representation of the plot that is based on pictorial information involves recoding processes (*recoding interference*). Future research is needed to determine the boundary conditions of cross-codal integration during narrative comprehension.

## Appendix

**Table 2:** Counterbalancing procedure of Experiment 1

		Bridging event		
Counterbalancing	Story	1	2	3
1	1	Picture	Absent	Text
	2	Picture	Text	Absent
	3	Absent	Picture	Text
	4	Absent	Text	Picture
	5	Text	Picture	Absent
	6	Text	Absent	Picture
2	1	Picture	Text	Absent
	2	Absent	Picture	Text
	3	Absent	Text	Picture
	4	Text	Picture	Absent
	5	Text	Absent	Picture
	6	Picture	Absent	Text
3	1	Absent	Picture	Text
	2	Absent	Text	Picture
	3	Text	Picture	Absent
	4	Text	Absent	Picture
	5	Picture	Absent	Text
	6	Picture	Text	Absent
4	1	Absent	Text	Picture
	2	Text	Picture	Absent
	3	Text	Absent	Picture
	4	Picture	Absent	Text
	5	Picture	Text	Absent
	6	Absent	Picture	Text
5	1	Text	Picture	Absent
	2	Text	Absent	Picture
	3	Picture	Absent	Text
	4	Picture	Text	Absent
	5	Absent	Picture	Text
	6	Absent	Text	Picture
6	1	Text	Absent	Picture
	2	Picture	Absent	Text
	3	Picture	Text	Absent
	4	Absent	Picture	Text
	5	Absent	Text	Picture
	6	Text	Picture	Absent

## Footnotes

1 The authors thank Joe Magliano and G. A. Radvansky for generously sharing their stimulus material.
